# Opiorphin as a biomarker of orofacial conditions: a meta-analysis

**DOI:** 10.1038/s41598-023-42051-y

**Published:** 2023-09-19

**Authors:** André Luís Porporatti, Claudia Aparecida de Oliveira Machado, Ivan Alajbeg, Iva Z. Alajbeg, Elzbieta Paszynska, Monika Dmitrzak-Weglarz, Adeline Braud, Yves Boucher

**Affiliations:** 1 Laboratoire de Neurobiologie OroFaciale (LabNOF, EA7543), Service Odontologie, Université Paris Cité, Groupe Hospitalier Pitié Salpêtrière-APHP, 47-83 Bd de l’Hôpital, 75651 Paris Cedex 13, France; 2 GHPS Assistance Publique Hôpitaux de Paris, Paris, France; 3 Federal University of Uberlandia, Uberlandia, Brazil; 4 Oral Medicine, School of Dental Medicine, University of Zagreb, Zagreb, Croatia; 5 Prosthetic Dentistry, School of Dental Medicine, University of Zagreb, Zagreb, Croatia; 6 Department of Integrated Dentistry, Poznan University of Medical Sciences, Poznan, Poland; 7 Department of Psychiatric Genetics, Department of Psychiatry, Poznan University of Medical Sciences, Poznan, Poland

**Keywords:** Diagnostic markers, Oral diseases

## Abstract

The aim of this meta-analysis was to answer the following question: “Are there any differences in opiorphin biomarker concentrations between different orofacial conditions and controls?”. Two reviewers searched for observational studies that evaluated the levels of opiorphin in orofacial conditions, annotated in seven main databases and three that compile gray literature. Of the 443 articles obtained initially, 8 met the inclusion criteria for quantitative analyses. Relative percentages showed a mean 24.1% higher opiorphin concentration in chronic conditions (Burning Mouth Syndrome, Oral Potentially Malignant Diseases and Temporomandibular Disorder) compared to controls; 33.2% higher opiorphin in sustained pain (Symptomatic Irreversible Pulpitis, Symptomatic Apical Periodontitis, Painful Oral Soft-tissue conditions); and 21.7% higher opiorphin after stimuli (Corneal Foreign Body, Capsaicin). Meta-analysis revealed a standardized mean difference of 0.62 [0.02, 1.22] in the absolute concentration of opiorphin in saliva for the chronic group compared to the control. The analogous values for the sustained group and the stimulated group were 2.24 [0.34, 4.14] and 0.43 [0.00, 0.85], respectively. No differences in opiorphin levels were found for ‘after Local Anesthesia before Tooth Extraction’ or for apicoectomy. Based on the available evidence, in general, a statistically higher level of opiorphin is found in orofacial conditions. Salivary opiorphin levels are elevated in chronic, persisted and acute pain conditions, presumably reflecting a physiological homeostatic adaptative response to different conditions such as stress or pain. Salivary opiorphin might therefore be used as a valuable biomarker in several oral disorders.

## Introduction

Orofacial pain (OP) defined as pain perceived in the face and/or oral cavity is caused by diseases or disorders of regional structures, by dysfunction of the nervous system, or through referral from distant sources^1^. This prevalent condition affects approximatively 20% of the population^2^, and encompasses a range of diagnoses^3^. These include acute and chronic pain, with the most prevalent being dental, periodontal and mucosal pain, temporo-mandibular disorders, primary or secondary burning mouth, painful trigeminal neuropathies. Similar to spinal pain, but with specificities related to the trigeminal system, OFP can be inflammatory, neuropathic or nociplastic in nature. OFP diagnosis is difficult, involving many health care specialists and often necessitating additional diagnosis tools like imaging and biological tests. Difficult also is its management, especially for chronic pain. As a consequence, OFP is a burden impacting quality of life of individuals and entailing considerable societal financial costs. Oral diseases affect more than 3.5 billion people worldwide with dental caries, frequently associated with pain, being the most prevalent disease^4,5^. Direct treatments for oral diseases account for approximatively 4.6% of global health expenditures^6^. Regarding persistent orofacial pain only, Breckons et al.^7^ estimated that mean out-of-pocket costs per person over a 6-month period were £333, with indirect costs reaching £1242.

Efforts to diagnose and treat OFP would benefit from the development of biomarkers in the context of the various inflammatory, infectious, autoimmune, and premalignant/malignant conditions that can affect the orofacial region. Saliva offers numerous advantages as a source of biomarkers of orofacial (and other) abnormalities, including pain^8–10^. For example, saliva and its constituents have been under investigation for more than a century, and collecting saliva samples is non-invasive and convenient^11–13^.

Opiorphin, a newly discovered pentapeptide (Gln–Arg–Phe–Ser–Arg) present in human saliva, exhibits analgesic and anxiolytic effects^14^ with promising therapeutic applications. It was first isolated by a dual biochemical and functional approach^14^, based on two related peptides, spinorphin and sialorphin, isolated from bovine spinal cord^15^ and rat saliva, respectively^16,17^. Opiorphin acts as an inhibitor of zinc metalloectopeptidases (MZPs), a class of proteases including neutral endopeptidase and aminopeptidase-N^14^. These membrane-anchored enzymes degrade circulating peptides such as enkephalins and substance P to limit their physiological roles. Therefore, MZP inhibition prolongs the physiological effects of natural peptides and, interestingly in therapeutics, it avoids the side effects observed with drugs acting as receptor agonists. For example, in vitro, opiorphin completely protects Met-enkephalin from degradation without directly interacting with opioid receptors. Opiorphin produces antinociceptive and anxiolytic/antidepressant effects with no associated tolerance (or morphine cross-tolerance)^18–20^ and displays analgesic properties similar to those of morphine in an acute mechanical pain model in rats^14^. Recent translational studies have shown that a synthetic analog of opiorphin suppresses mechanical hypersensitivity in a rat model of neuropathic pain^21^. Opiorphin’s effects are suppressed by the opioid receptor antagonist naloxone, evidencing its action through endogenous opioid-dependent pathways^20^. Taken together, these data suggest a promising role for opiorphin as a biomarker as well as a therapeutic agent.

While the physiological cycle of opiorphin remains unclear, the rat analog sialorphin is secreted in salivary mandibular glands in response to diverse conditions like fear, stress, or pain, serving as a molecular effector within the cervical sympathetic trunk submandibular gland (CST-SMG) axis^22^. This axis modulates homeostatic processes and underscores salivary glands as a source of locally and systemically active immunoregulatory and anti-inflammatory factors^22^. Given that many OFP conditions are influenced by inflammatory factors^23–26^ exploring the CST-SMG axis through opiorphin release could enhance the understanding and management of OFP conditions.

In humans, several studies have assessed variations of salivary opiorphin levels under different conditions including pain and stress^27,28^. Opiorphin's potential as a biomarker has been explored in various pathologies such as Temporomandibular Disorder (TMD)^27^, Burning Mouth Syndrome (BMS)^29,30^, Oral Potentially Malignant Disorders (OPMD)^8^, dental pain as Symptomatic Irreversible Pulpitis (SIP)^31^ and Symptomatic Apical Periodontitis (SAP)^31^, anorexia nervosa^32,33^, ocular pain with Corneal Foreign Body (CFB)^34^, and depressive^19^ and erectile disorders^35–37^. However, the medical literature's results are yet to be fully integrated, sometimes showing apparent contradictions across conditions. For instance, opiorphin levels have been reported to increase in dental inflammatory pain^31^, decrease after oral local anesthesia^38^. In BMS, perhaps the most emblematic conditions for looking a link between opiorphin and oral pain, several measurements have shown conflicting results. For example Ruangsri et al.^39^ report a decrease of opiorphin levels in BMS patients compared to control subjects when Salaric et al.^29,30^ report an increase and Boucher et al.^29,30^ a statistically non-significant decrease. Heterogeneity in methodology and study quality might contribute to these discrepancies, highlighting the need for a comprehensive review of available evidence concerning OFP and opiorphin release.

To our knowledge, there is no systematic review or meta-analysis aimed at measuring salivary opiorphin levels in orofacial conditions. Therefore, based on these premises, the aim of this meta-analysis was to answer the following question: “Are there any differences in opiorphin biomarker concentrations between different orofacial conditions and controls?”. Our hypothesis was that higher levels of opiorphin are founded in pain conditions than controls In case of differences, the surrogate questions are “can methodological differences account for discrepancies in the studies?”, “are there any differences in salivary opiorphin levels according to OFP subtypes?”. “is there any correlation between opiorphin salivary levels and intensity of OFP”, and “what is the time course of opiorphin release in OFP?”.

## Materials and methods

### Protocol and registration

This systematic review conforms to the Preferred Reporting Items for Systematic Reviews and Meta-Analyses (PRISMA) Checklist^40^. The protocol is registered in the International Prospective Register of Systematic Reviews (PROSPERO) under number CRD42021269661.

### Eligibility criteria

We included observational studies (case–control, cross-sectional and cohort studies) and case series (> 10 cases) that evaluated opiorphin in orofacial conditions, but included only adults (18–65 years old). The included studies evaluated opiorphin extracted from blood (including plasma), saliva (different preparations), urine, or tears, assessed by ELISA or chromatography.

Overall, the inclusion criteria were based on the PECOS question^41^:

Population (P): Humans; Exposure (E): Opiorphin; Comparison (C): Controls; Outcome (O): Different concentrations; Study design (S): observational studies and case series. No data, sex or language restrictions were applied to the search strategy.

The exclusion criteria were as follows:

(1) Studies in animals; (2) studies where no orofacial condition was evaluated; (3) studies where opiorphin was not evaluated through saliva, blood, urine or tears; and (4) literature reviews, intervention studies, books, letters, case reports (< 10 cases) and personal opinions.

### Information sources and search strategy

Detailed individual search strategies were developed for each bibliographic electronic database: Cochrane, EMBASE, Latin American and Caribbean Health Sciences (LILACS), LIVIVO, PubMed (including Medline), Scopus and Web of Science. A gray literature search was performed on Google Scholar, Open Grey and ProQuest. All database searches were conducted from the starting coverage date through October 28, 2022. More information on the search strategies is provided in Appendix 1 (which can be found online). Furthermore, the authors hand-searched the reference lists of the selected articles for any additional references that might have been missed in the database searches. We also sought out articles by contacting relevant experts. These individuals were contacted if they had published 2 or more papers about the same systematic review topic. All references were managed and the duplicated hits were removed by using reference manager software (EndNote X7^®^ Basic-Thomson Reuters, New York, EUA).

### Selection process and data collection process

This part followed a two-phase process. In phase-one, two authors (A.L.P. and C.A.O.M) independently evaluated the titles and abstracts of all identified electronic database citations. In phase-two, the same authors evaluated full-text data. They independently screened papers at phase-one and -two, applied the eligibility criteria, collected key information from the selected studies, and crosschecked the information. The final selection was based solely on full-text assessment of the studies. When disagreement arose, a third author (Y.B.) was involved to make a final decision about whether to include or exclude a study.

### Data items

For each of the included studies, the following items were recorded: author(s), year of publication, country, sample size, demographic features of the sample (n, mean age and standard deviation, percentage of women), method of collection, information about this method, results, and main conclusions. When the required data were not complete, the reviewers (A.L.P. and C.A.O.M) attempted to contact the study authors to retrieve any unpublished information. Three attempts were made in a 30-day period, by email for the first, second and last author.

### Study risk of bias assessment

The methodological quality of each included observational study was evaluated through Joanna Briggs Institute (JBI) Critical Appraisal Tools to assess risk of bias^42–44^. The answers could be “yes”, “unclear”, “no”, or “not applicable”. Decisions about scoring were agreed upon by all reviewers before critical appraisal commenced. The same two reviewers (A.L.P. and C.A.O.M) worked out any initial differences regarding data analysis. A third author (Y.B.) was involved to reach a decision in case of uncertainty. After these ratings, the risk of bias was categorized according to: (1) low risk of bias, if all criteria were met, (2) unclear risk of bias, if one or more criteria were not described exactly how they were met, and (3) high risk of bias, if one or more criteria were not met^44^. Figures of the quality assessment of all included studies were generated with Review Manager 5.3 (RevMan 5.3, The Nordic Cochrane Centre, Copenhagen, Denmark)^45^.

### Effect measures

We considered the results in terms of both absolute and relative differences in fluid opiorphin concentrations. The standardized mean difference was used as an effect measure for continuous outcomes. To standardize the results of the studies to a uniform scale, we transformed all measures to ng/ml values. Any type of outcome measurement was considered. We attempted to standardize the measurements as mean and standard deviation (SD).

### Synthesis methods

Statistical pooling of data using meta-analysis was carried out where studies were considered combinable and relatively homogeneous in relation to design, interventions, and outcomes. Heterogeneity within studies was evaluated either by considering clinical (differences about participants, type of interventions and results), methodological (design, and risk of bias) and statistical characteristics (effect of studies) or by using the inconsistency index (I^2^) statistical test^45^.

If quantitative synthesis was appropriate, analysis of the standardized mean difference was performed using RevMan 5.3, and heterogeneity was assessed using the Cochran Q test and I^2^ statistics. For the analysis model, a fixed or random effect was based on an expectation of whether the intervention effects were truly identical, preferring the fixed-effect model if this was likely and a random-effects model if this was unlikely. Heterogeneity was calculated by I^2^, and a value greater than 50% was considered an indicator of substantial heterogeneity between studies. The significance level was set at 5%. The meta-analysis was performed with the aid of Review Manager software version 5.3.5 (Nordic Cochrane Center, Copenhagen, Denmark) for continuous data following the appropriate Cochrane Guidelines^45^.

We also considered generating a funnel plot as a graphic to address reporting biases, but in the end our sample size was too small (< 10 articles) for that method of analysis.

### Risk of bias across studies and reporting bias assessment

The risk of bias across studies was considered in terms of an overall risk the study results may present, which could influence meta-analysis data. Methodological and statistical heterogeneity was evaluated by comparing the variability in study designs and the risk of bias. Furthermore, we also assessed the risk of bias due to missing results.

### Certainty assessment

A summary of the overall strength of evidence available was presented using "Grading of Recommendations Assessment, Development and Evaluation" (GRADE) Summary of Findings (SoF) tables, using GRADEpro software^45^.

### Ethical approval

This article does not contain any studies with human participants or animals performed by any of the authors.

### Informed consent

For this type of study, formal consent is not required.

## Results

### Study selection

**Figure 1:**
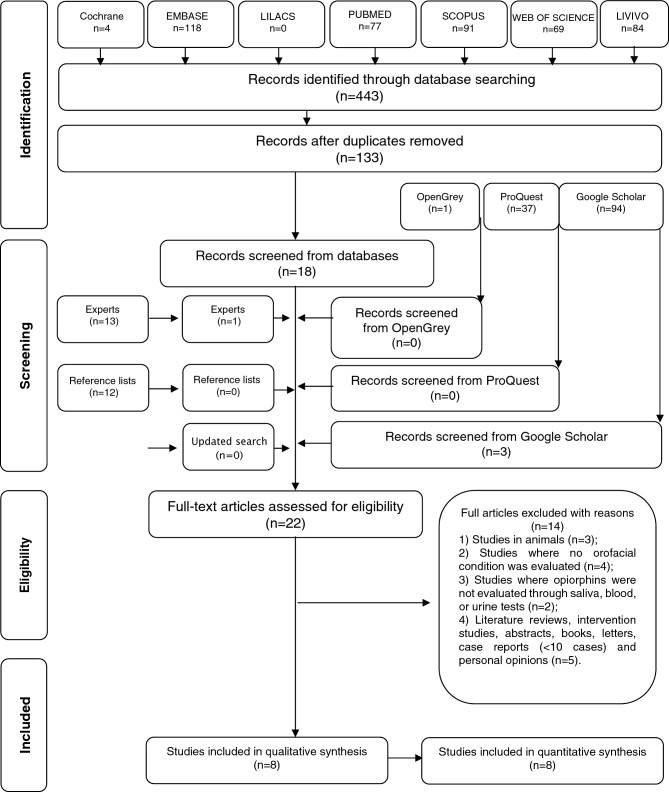
Flow diagram of the literature search and selection criteria. Adapted from PRISMA.

Our initial database searches up to June 2021, identified 443 studies. After eliminating duplicated hits, 133 studies remained of which 115 were excluded after title and abstract review, resulting in 18 articles. In addition, 71 studies were found with Google Scholar, 1 with OpenGrey, and 37 with ProQuest. Of these latter 109 studies, 3 from Google Scholar were selected for full-text reading. No additional study was selected following hand-searching of the reference lists of the included studies, although 1 further study was included based on suggestion by an expert. Thus, 22 studies became part of phase-2. The search was updated on October 28, 2022. We found a total of 103 more papers (9 in PubMed, 12 in Scopus, 1 in Cochrane, 43 in Web of Science, 0 in LILACS, 13 in EMBASE, 12 in LIVIVO, and 13 in Google Scholar); however, all 103 were excluded because “no orofacial condition was evaluated” (exclusion criterion #2). During phase-2, 14 of the 22 studies were excluded (reasons for exclusion are given in Appendix 2), leaving 8 studies for qualitative and quantitative synthesis. A flowchart of the process of identification, inclusion and exclusion of studies is shown in Fig. 1.

### Study characteristics and results of individual studies

In the 8 studies evaluated, mean sample size ranged from 22^38^ to 144^46^, with a total of 338 subjects with one orofacial condition, and 118 healthy controls. The proportion of women in the studies ranged from 40^31^ to 90.4%^30^. Studies were conducted in Croatia^27,29^, France^30^, India^8,46^, Iraq^38^, and Turkey^31,34^. All studies were published in English. The study by Alajbeg et al.^27^ was part of a clinical trials protocol, and the data were obtained by contacting the authors by e-mail.

Studies encompassed different orofacial conditions: Chronic Temporomandibular Disorder (TMD)^27^, Burning Mouth Syndrome (BMS)^29,30^, Painful Oral Soft-tissue Conditions (POSC)^8^, Oral Potentially Malignant Disorders (OPMD)^8^, Symptomatic Irreversible Pulpitis (SIP)^31^, Symptomatic Apical Periodontitis (SAP)^31^, Corneal Foreign Body (CFB)^34^, Local Anesthesia before Tooth Extraction (LA)^46^ and Apicoectomy^38^.

Study designs included 5 case–control studies^8,27,29,30,34^, 1 randomized clinical trial^46^ and 2 quasi-randomized studies^31,38^. Opiorphin levels were measured in saliva in 7 studies, except Boucher et al.^30^ also tested blood and urine; Ozdogan et al.^34^ measured opiorphin in tears. A human opiorphin ELISA kit was used in all but 2 studies; the exceptions were Alajbeg et al.^27^ and Saláric et al.^29^ who performed electrospray positive ionization-mass spectrometric multiple reaction monitoring (ESI+/MRM). Table 1 and Appendix 6 (descriptive methods for opiorphin collection) summarize the descriptive characteristics of the included studies.

**Table 1:** Summary of descriptive characteristics of the included articles (n = 8).

Author Year Country	Orofacial condition	Study design	Sample groups (n) Mean age (SD) % female	Biological fluids for collection	Extra data	Results (in ng/ml)	Main conclusion
Alajbeg I 2021 Croatia CLINICAL TRIAL PROTOCOL	Chronic TMD	Case–control	TMD 11 NR	Saliva	Intensity of burning pain sensation	TMD 1.890066 (1.296292) 1.995090 (1.945315) 2.277035 (2.052423)	Statistically higher levels of opiorphin at baseline were observed in chronic TMD patients, compared to control group
			Healthy 14 NR				
						Control I. 0.646542 (0.467077) II. 0.794426 (0.650741) III. 0.700216 (0.520407)	
Al Saffar M 2013 Iraq	Local anesthesia after tooth apicoectomy	Quasi-experimental (before and after)	22 63.6%F 25.5 ± 6.68 years for female and 29.5 ± 3.5 years for male with age range 20–40	Saliva (unstimulated)	5–7 min after anesthesia, pain intensity using VAS was measured	Before anesthesia 5.96 ± 5.38	A significant effect of local anesthesia on opiorphin salivary levels Opiorphin level before and after administration of local anesthesia ranged between (5.96–14.49) ng/ml within the normal range of salivary opiorphin (2.8–25.9) ng/ml
						After anesthesia 14.49 ± 3.66	
Boucher Y 2016 France	iBMS	Case–control	iBMS 21 58.5 (11.7) 90,4% F	Saliva (basal and stimulated) 2 ml	HADS	iBMS Basal saliva (37.8 ± 42.5) Saliva stimulated (28.8 ± 25.3) Blood (4.6 ± 5.4) Urine (68.5 ± 259.8)	Basal and stimulated salivary opiorphin levels of iBMS patients and control subjects were not significantly different neither for the whole sample nor for the female subgroup nor between subgroups of age (≤ 60 years old and > 60 years old). Concentration of blood opiorphin was significantly higher in iBMS patients than in control subjects (4.5 ± 5.4 vs 1.8 ± 1.4 ng/mL) especially when regarding female subjects (5.1 ± 5.6 ng/ml for iBMS women and 2.1 ± 1.4 ng/ml for healthy women; p ≤ 0.05, n = 19)
				Blood 30 ml, and			
				Urine 20 ml			
			Control 21 58.9 (11.5) 90.4% F				
						Control Basal saliva (67.6 ± 188.9) Saliva-stimulated (31.1 ± 29.1) Blood (1.9 ± 1.4) Urine (8.9 ± 6.2)	
Nejad N 2020 India	Painful oral soft-tissue conditions such as traumatic ulcer, recurrent aphthous ulcer, oral candidiasis, OPMD such as lichen planus, oral submucous fibrosis, carcinoma of oral cavity, and BMS	Case–control	Controls 20 36 (2.7) NR	Saliva	Systemic condition and presence of deleterious habits such as alcohol, tobacco use, and pain history	Control 7.108 ± 2.535	No significant correlation was found between opiorphin levels, VAS, and HADS score. However, a positive correlation was observed between salivary opiorphin levels and age of the patient (r = 0.028)
						Traumatic and inflammatory conditions 9.409 ± 2.369	
			Traumatic and inflammatory conditions of oral mucosa 20 36 (2.7) NR				
					HADS questionnaire was also used		
						OPMDs 8.268 ± 2.414	
			OPMDs and oral cancer patients 20 36 (2.7) NR				
Ozdogan M 2019 Turkey	SIP SAP	Quasi-experimental (before and after)	SIP 15 32.64 (14.23) 40% F	Saliva	Measuring the pain levels, using a VAS-10 cm	Pre-Treatment SIP 37.66 ± 6.15 SAP 28.78 ± 5.81 7 days after SIP 20.30 ± 2.82 SAP 20.47 ± 2.67 30 days after SIP 18.74 ± 1.15 SAP 18.58 ± 1.85	Saliva opiorphin levels increase significantly in inflammation-related dental pain. Also, a strong correlation was observed between the reported level of pain and the saliva opiorphin level
			SAP 24 39.04 (12.24) 46% F				
Ozdogan S 2020 Turkey	Corneal foreign body	Case–control	Control 34 31.5 NR	Tears	Measurement of pain levels using a VAS-10 cm	Corneal foreign bodies 0.13483 ± 0.06027	Acute pain caused by corneal foreign objects causes an increase in tear opiorphin levels. No correlation between the level of reported pain and tear opiorphin levels was found
						Control 0.10980 ± 0.03724	
			Corneal foreign bodies 32 38.5 NR				
Parida S 2017 India	Local anesthesia after tooth extraction	Randomized clinical trial	The 144 patients were divided into four groups of 36 patients	Saliva	None	The salivary opiorphin levels for all patients ranged from 0.8 to 9.3 ng/ml before administration of local anesthesia (mean 4.8 ng/ml). After administration of local anesthetic, the salivary opiorphin levels were found to be between 0.9 and 9.1 ng/ml (mean 4.6 ng/ml). The difference was not statistically significant (p < 0.05) The mean rise of salivary opiorphin level was 0.28 ng/ml with local infiltration and 0.4 ng/ml with the inferior alveolar nerve block	This study did not show much association between various local anesthetic agents and techniques and change in salivary opiorphin levels
			Age of the patients ranged from 20 to 65 years 50% F				
			Group 1 Inferior alveolar nerve block				
			Group 2 Local infiltration,				
			Group 3 Infraorbital nerve block				
			Group 4 Posterior superior alveolar nerve block				
			In each group, 12 patients each were randomized to receive either lignocaine, articaine or bupivacaine				
Saláric I 2016 Croatia	Burning Mouth Syndrome	Case–control	BMS 29 67.45 (9.44) 83% F	Saliva	Periodontal health was assessed by papillary bleeding index (PBI) on Ramfjord index teeth (nos. 3, 9, 12, 19, 25, and 28). When a subject was missing a Ramfjord index tooth, a tooth closest to it was assessed	BMS UWS 8.129 ± 6.445	There was no statistically significant difference neither in age (t test, t = 0.048, p = 0.962) nor in gender (χ^2^ test, χ^2^ = 1.507, p = 0.220) between the two groups Differences between UWS and SWS within groups were also not statistically significant
						SWS 5.819 ± 3.594	
			Control 29 67.31 (12.66) 69% F				
						Control UWS 5.017 ± 2.585	
						SWS 4.992 ± 3.212	

*ELISA* Enzyme Linked ImmunoSorbent Assay, *F* female, *HADS* hospital anxiety and depression scale, *iBMS* Idiopathc Burning Mouth Syndrome, *NR* not reported, *OPMDs* oral potentially malignant disorders, *SAP* symptomatic apical periodontitis, *SIP* symptomatic irreversible pulpitis, *SWS* stimulated whole saliva, *TMD* temporomandibular disorders, *UWS* unstimulated whole saliva, *VAS* Visual Analogue Scale.

### Risk of bias in studies

**Figure 2:**
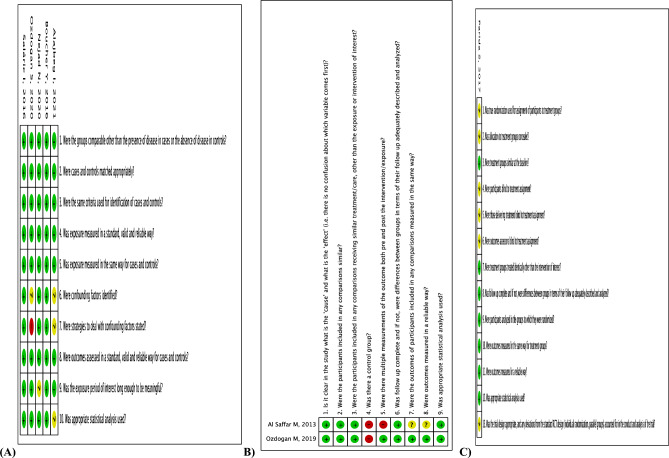
JBI Critical Appraisal Tools to assess risk of bias summary in (**A**) case–control studies; (**B**) quasi-experimental studies; and (**C**) randomized clinical trials.

Risk of bias was heterogeneous among the 8 studies. Using JBI Critical Appraisal Tools, 2 studies were classified as having low risk of bias^29,30^, 3 as unclear^8,27,46^, and 3 as high risk of bias^31,34,38^. The higher risk of bias related to strategies to deal with confounding factors. The complete item list is presented in Fig. 2 and Appendix 3.

### Results of syntheses

Individually, TMD, SIP, SAP and CFB were associated with higher concentrations of opiorphin than the control, whereas BMS, POSC, OPMD and LA showed no difference. We further divided the conditions into 4 groups: *chronic orofacial* group (TMD, BMS and OPMD); *sustained pain* group (SIP, SAP and POSC); *acute pain after local anesthesia* group (tooth extraction and apicoectomy); and *stimulated* group (after CFB, and capsaicin), according to the physio-pathological processes underlying these heterogenous conditions. TMD, BMS and OPMD are chronic conditions (> 3 months) with peripheral and central involvement. SIP and SAP are similar conditions involving long-term alterations in nerve pathways (bacterial inflammation and sensitization) which peak in acute pain, but are not considered as chronic pain conditions. Regarding the others, we thought of merging the *acute pain* and *stimulated* groups but the studies are fundamentally different in nature. CFB and capsaicin provoke pain on a short-term basis (minutes or hours), whereas the anesthesia study aimed to suppress pain with an anesthetic.

Based on the data presented in the included studies, we calculated the relative percentage of opiorphin change versus the control group. In the chronic group, patients with TMD exhibited a 65.8% (SD 68.7%) higher opiorphin concentration than controls. An increase was also found for BMS, with 38.3% (SD 79.3%), and OPMDs, with 24.5% (SD 25.2%). In the sustained group, SIP exhibited 50.2% (SD 16.3%) and SAP 35.4% (SD 20.2%) higher opiorphin concentrations before endodontic treatment; and POSC was 14.0% higher (SD 29.2%). After anesthesia, opiorphin levels were 143.1% (SD 90.27%) higher in apicoectomy subjects, and 4.2% lower in tooth extraction subjects. After stimulation, opiorphin was 22.9% (SD 27.6%) and 20.5% (SD 68.7%) higher in cases of CFB and after capsaicin, respectively (Appendix 4). Overall, the relative opiorphin concentration was 24.1% (SD 60.9%) higher in chronic conditions compared to controls; 33.2% (SD 21.9%) higher in persisted pain, and 21.7% (SD 56.7%) higher after stimuli. No differences were found after local anesthesia.

**Figure 3:**
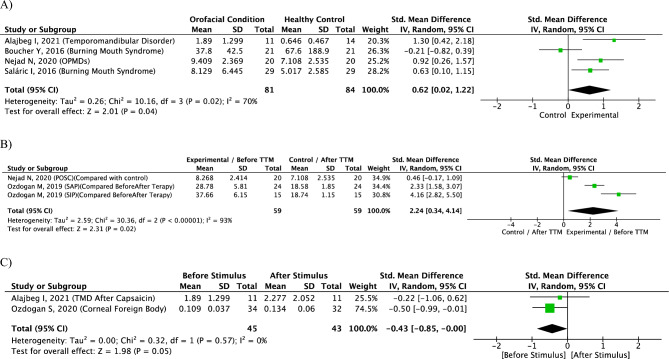
Forest plots indicating different concentration of opiorphins in saliva on orofacial pain conditions compared to controls. Graphs generated with Review Manager 5.3 (RevMan 5.3, The Nordic Cochrane Centre, Copenhagen, Denmark). (**A**) Chronic orofacial group (TMD, BMS and OPMD); (**B**) sustained pain group (SIP, SAP and POSC); (**C**) stimulated group (after CFB, and capsaicin). *CI* confidence interval, *OPMDs* oral potentially malignant disorders, *POSC* painful oral soft-tissue conditions, *SAP* symptomatic apical periodontitis, *SIP* symptomatic irreversible pulpitis, *SD* standard deviation, *TTM* treatment.

In addition, we conducted a meta-analysis of the 8 selected studies (Fig. 3). To minimize bias, we used the standardized mean difference as a measure of effect size, because the studies all assess the same outcome but measure it in a variety of ways. The heterogeneity between the studies was high on this meta-analysis (I^2^: 70–90%) because the results were derived from different types of orofacial conditions, and a random effect was considered. Meta-analysis of the chronic group (TMD, BMS, OPMDs) showed a 0.62 [0.02, 1.22] standardized mean difference in the absolute concentration of opiorphin in saliva compared to controls. The sustained group (painful oral soft-tissue conditions vs. controls; and SAP and SIP, before vs. after treatment) showed a 2.24 [0.34, 4.14] standardized mean difference. The stimulated group (capsaicin, CFB) showed a 0.43 [0.00, 0.85] standardized mean difference in the absolute concentration of opiorphin ‘after stimulus’ when compared to ‘before stimulus’. No meta-analysis was feasible for the *acute pain after local anesthesia* group, owing to a lack of SD data. In general, a statistically higher level of opiorphin was observed in orofacial conditions compared to controls.

### Risk of bias across studies and reporting biases

The 8 selected studies had case–control, randomized and quasi-randomized designs. The main methodological problem concerned assessment of outcomes in a healthy control group. Moreover, strategies to deal with confounding factors such as age, sex and psychosocial status were not always addressed. The main problem related to reporting biases was a lack of standardization of units of measure; for example, in some studies the values were only presented in figures and not as precise numerical values in tables.

### Certainty of evidence

The overall quality of evidence identified using GRADE’s SoF tables was assessed as very low (Appendix 5), because of high risk of bias, inconsistency (I^2^) greater than 70%, outcomes not related to the review question, and small pooled sample size.

## Discussion

To our knowledge, this study is the first to systematically review the available evidence related to the concentration of opiorphin in patients with orofacial conditions compared to control subjects. Opiorphin levels were overall increased in OFP conditions. Relative percentages showed 24.1%, 33.2% and 21.7% higher opiorphin levels in chronic pain (TMD, BMS, and OPMDs), sustained pain (SAP, SIP, POSC), and after painful stimulus (CFB, capsaicin), respectively. Meta-analysis found significant standardized mean differences in the absolute concentration in all pain groups compared to controls. While interesting, these findings should be interpreted with caution since several factors may limit their value, as discussed below.

### Technical issues

Opiorphin levels were not assessed by the same method in all 8 studies, potentially leading to differences in absolute values. Two studies used HPLC coupled to ionization^27,29^ to measure opiorphin and 6 used ELISA^8,31,34,38,46,47^, with 4 different analysis kits. This might explain some discrepancy in the results. ELISA is easy to perform and relatively inexpensive but has a relatively high Limit of Detection (LOD), i.e., the smallest amount of the analyte that can be detected in the test sample. Electrospray positive ionization-mass spectrometric multiple reaction monitoring (ESI+/MRM)^29,48,49^ provides much higher sensitivity and specificity but requires highly specialized equipment and software, which limits its usefullness.

### OFP subtypes

The studies included in this review encompass different orofacial conditions. Individually, TMD, SIP, SAP and CFB were reported to have higher concentrations of opiorphin than controls; however, no differences were noted for BMS, POSC, OPMD and LA. When considering the subcategories i.e. chronic pain, sustained pain, acute pain after local anesthesia, and stimulated acute pain, comparative analysis suggest that chronic pain conditions result in higher opiorphin levels, although not for all conditions since BMS data do not support this finding. Indeed two studies report contradictory results^29,30^. The study of Boucher et al.^29,30^ reported non-significant differences in the concentration of opiorphin in controls compared to patients with BMS, whereas Salaric et al.^29,30^ found a higher concentration in the BMS group. In addition, the results of these studies slightly differed when the saliva collected for analysis was stimulated saliva or non-stimulated saliva. Furthermore another study, not included in this meta-analysis^39^ because only published in an abstract form, reported lower levels of salivary opiorphin in BMS patients. Overall, these discrepancies do not provide strong evidence for a link between opiorphin and BMS which might be related to the complex physiopathology of BMS. This condition is understood as a nociplastic condition including hormonal and neuropathic alterations, possibly related to stress^50^ when the other OFP included in this review display stronger nociceptive/inflammatory components. Besides chronic conditions, sustained pain conditions also produced an increase of opiorphin, reinforcing the hypothesis that opiorphin is produced as a long-term adaptive response. However; it must be emphasized that some studies are characterized by a high risk of bias owing to lack of clinical information. For example, in the OPMD study^8^ it is not clear whether BMS subjects were included.

Ozdogan et al.^31^ measured opiorphin levels in pain-free patients 30 days after endodontic treatment, effectively a control group; the opiorphin levels returned to normal after a sustained rise elicited by pain of pulpitis or periapical periodontitis. This likely reflects a long term process even if the pain peaks for just one or a few years. Indeed, studies with local anesthesia, including subjects before tooth extraction^46^ and apicoectomy^38^, showed no decrease of opiorphin after a few minutes or after one week, suggesting again sustained, long-term opiorphin production. For acute pain, measurements of opiorphin a few minutes after local anesthesia gave contradictory results: one study showed an increase in opiorphin levels of 143.1%^38^, whereas a second study reported a decrease of 4.1%^46^.

### Intensity of pain and opiorphin levels

A correlation between pain intensity on a visual analog scale (VAS) and opiorphin level was supported in only one study. Ozdogan et al.^31^ observed a positive correlation in the painful SIP and SAP pre-treatment group. Other studies did not find or did not report this parameter. However, it must be emphasized that these data are at high risk of bias. For instance, Al-Saffar et al.^38^ claimed an inverse correlation between opiorphin levels and VAS post-LA but provided no numeric pain evaluation before anesthesia, reporting only “painful patients” in need of apicoectomy. The same flaw in study design was found in the study of Parida et al.^46^ where no pain scores were measured in patients needing tooth extraction. Based on this literature, we could not find an association between local anesthesia and changes in opiorphin levels, and we could not extrapolate the results for direct association between VAS scores and opiorphin level. Further studies are necessary to document this association.

### Time course of opiorphin release

Alajbeg et al.^27^ stimulated the oral mucosa of subjects with capsaicin, and detected no opiorphin release in control subjects but in TMD patients. Although not yet published, this is the first study to document acute release of opiorphin in response to a painful stimulus in humans which seems to occur only in certain conditions. The study of Ozdogan et al.^34^ also supports the release of opiorphin after a CFB painful stimulus, although with a different time course (hours vs. minutes). It must also be mentioned that local anesthesia, before silencing peripheral nerves, is often accompanied by a pricking pain due to the needle insertion which may also generate stress, and could explain contradictory results. Therefore, more studies related to the time course of opiorphin release after nociceptive stimulation are needed.

Taken together, as the conditions reviewed here include mainly painful and stressful conditions, the data suggest that opiorphin is released in response to pain and/or stressful situations; interestingly, the only study to report a pathological non-painful condition, i.e. oral potentially/malignant conditions^8^, reported no significant increase of salivary opiorphin levels, thus supporting this assertion.

As a consequence, administration of opiorphin or its analogs might be useful in therapeutics. Indeed, studies suggest an analgesic effect of administration of a dual enkephalinase inhibitors, in animals’ models of ocular pain^51^ and migraine^52^.

### Influence of different factors on opiorphin release

Various factors have been described that can influence opiorphin levels, such as age and sex^30,53^, systemic health, use of medications, the most stimulated salivary gland^29^, the body fluid from which opiorphin is collected^30^ and psychosocial profile^16^.

Evidence is already available for higher concentrations of opiorphin in males compared to females^53^; in younger healthy adults (mean age 26 ± 6 years)^53^ compared to older ones with BMS (59 ± 12 years)^30^; in non-pregnant volunteers compared to sixth-month pregnant^53^; in unstimulated saliva secreted mainly by the submandibular glands compared to stimulated saliva, which is secreted mainly by the parotid glands^29^; and in serum compared to saliva^30^. In addition, one study found no correlation between the levels of opiorphin and systemic conditions or drug consumption^29^. Another important point concerns whether and how the psychosocial status of the patient may alter the salivary opiorphin levels. Patients with anxiety may experience a more negative emotional response to pain and increased susceptibility to stress^54^. Furthermore, sialorphin increases under acute stress conditions in rats, suggesting that psychosocial status may influence opiorphin levels in human subjects; therefore, studies related to this topic, i.e., different stressful conditions, should be encouraged^16,55^.

### Future directions

The present review emphasizes the need for better study designs and improved clinical information. Multicentric designs should be favored to control for cultural differences. Confounding factors such as age, sex, systemic health, use of medications, the body fluid sampled and the psychosocial profile of patients should all be analyzed, as well as pain levels in control groups, and tests conducted before and after intervention. Initiative on Methods, Measurement, and Pain Assessment in Clinical Trials (IMMPACT) recommendations can provide supportive guidelines^56^.

This review highlights the lack of knowledge related to physiologic conditions of opiorphin release. Experimental studies in both acute and chronic conditions should be encouraged, as well as dissociating pain and stress effects on opiorphin release. Finally, trials with different types of nociceptive stimulations such as capsaicin can be considered in future studies in order to decipher the mechanisms of opiorphin release in acute and chronic pain.

## Conclusions

The results of the present review may not be generalized due to the aforementioned limitations of the included studies, the higher risk of bias in some studies regarding strategies to deal with confounding factors, and very low GRADE level of evidence. Based on the available evidence, this meta-analysis suggests that salivary opiorphin levels are elevated in chronic, sustained and acute pain conditions, reflecting a physiological homeostatic adaptative response to different conditions such as pain and psychic stress. Salivary opiorphin might therefore be used as a valuable biomarker in oral inflammation.

### Supplementary Information

Supplementary Information.

## Data Availability

All of the data, material and methods which support the results can be found in the article.

## References

[1] Williams ACC, Craig KD (2016). Updating the definition of pain. Pain.

[2] Porporatti, A., *et al*., *Prevalence of Orofacial Pain Conditions: An Umbrella Review of Systematic Reviews. PROSPERO 2022 CRD42022377910*. https://www.crd.york.ac.uk/prospero/display_record.php?ID=CRD42022377910.

[3] International Classification of Orofacial Pain, 1st edition (ICOP). Cephalalgia, 2020. **40**(2): 129–221. 10.1177/0333102419893823 32103673

[4] World Health Organization (2022). Global Oral Health Status Report: Towards Universal Health Coverage for Oral Health by 2030.

[5] Peres MA (2019). Oral diseases: A global public health challenge. Lancet.

[6] Listl S (2015). Global economic impact of dental diseases. J. Dent. Res..

[7] Breckons M (2018). DEEP study: Indirect and out-of-pocket costs of persistent orofacial pain. J. Dent. Res..

[8] Nejad NK, R.P., Kar A, Sujatha S. (2020). Quantitative analysis and expression of salivary opiorphin in painful oral soft-tissue conditions: A descriptive study. J. Glob. Oral Health.

[9] Fischer HP, Eich W, Russell IJ (1998). A possible role for saliva as a diagnostic fluid in patients with chronic pain. Semin. Arthritis Rheum..

[10] Jang MU (2011). Plasma and saliva levels of nerve growth factor and neuropeptides in chronic migraine patients. Oral Dis..

[11] Michela B (2021). Liquid biopsy: A family of possible diagnostic tools. Diagnostics (Basel).

[12] Khurshid Z (2021). Biochemical analysis of oral fluids for disease detection. Adv. Clin. Chem..

[13] Chojnowska S (2021). Salivary biomarkers of stress, anxiety and depression. J. Clin. Med..

[14] Wisner A (2006). Human Opiorphin, a natural antinociceptive modulator of opioid-dependent pathways. Proc. Natl. Acad. Sci. USA.

[15] Nishimura K, Hazato T (1993). Isolation and identification of an endogenous inhibitor of enkephalin-degrading enzymes from bovine spinal cord. Biochem. Biophys. Res. Commun..

[16] Rougeot C (1994). Selective processing of submandibular rat 1 protein at dibasic cleavage sites. Salivary and bloodstream secretion products. Eur. J. Biochem..

[17] Rougeot C (2003). Sialorphin, a natural inhibitor of rat membrane-bound neutral endopeptidase that displays analgesic activity. Proc. Natl. Acad. Sci. USA.

[18] Popik P (2010). Human opiorphin: The lack of physiological dependence, tolerance to antinociceptive effects and abuse liability in laboratory mice. Behav. Brain Res..

[19] Javelot H (2010). Human opiorphin is a naturally occurring antidepressant acting selectively on enkephalin-dependent delta-opioid pathways. J. Physiol. Pharmacol..

[20] Rougeot C (2010). Systemically active human opiorphin is a potent yet non-addictive analgesic without drug tolerance effects. J. Physiol. Pharmacol..

[21] Van Elstraete A (2018). The opiorphin analog STR-324 decreases sensory hypersensitivity in a rat model of neuropathic pain. Anesth. Analg..

[22] Mathison RD (2012). Autonomic regulation of anti-inflammatory activities from salivary glands. Chem. Immunol. Allergy.

[23] Sessle BJ (2011). Peripheral and central mechanisms of orofacial inflammatory pain. Int. Rev. Neurobiol..

[24] Shrivastava M, Battaglino R, Ye L (2021). A comprehensive review on biomarkers associated with painful temporomandibular disorders. Int. J. Oral Sci..

[25] Liu Q (2022). Transcriptional alterations of mouse trigeminal ganglion neurons following orofacial inflammation revealed by single-cell analysis. Front. Cell Neurosci..

[26] Korczeniewska OA (2022). Pathophysiology of post-traumatic trigeminal neuropathic pain. Biomolecules.

[27] Alajbeg, I., Oxidative stress and opiorphin in temporomandibular disorders (ROStrO-TMD). 2017: ClinicalTrials.gov Identifier: NCT03029494.

[28] Orabović I (2021). Salivary Opiorphins as a response to Capsaicin stimulation: A comparison of Temporomandibular Disorder patients and healthy controls. Acta Stomatol. Croat..

[29] Salarić I, Sabalić M, Alajbeg I (2017). Opiorphin in burning mouth syndrome patients: A case–control study. Clin. Oral Investig..

[30] Boucher Y (2017). Opiorphin levels in fluids of burning mouth syndrome patients: A case–control study. Clin. Oral Investig..

[31] Ozdogan MS (2019). Salivary opiorphin in dental pain: A potential biomarker for dental disease. Arch. Oral Biol..

[32] Paszynska E (2020). Is there a link between stress and immune biomarkers and salivary opiorphin in patients with a restrictive-type of anorexia nervosa?. World J. Biol. Psychiatry.

[33] Paszynska E (2020). Salivary opiorphin levels in anorexia nervosa: A case–control study. World J. Biol. Psychiatry.

[34] Ozdogan S (2020). Tear opiorphin levels in ocular pain caused by corneal foreign body. Cornea.

[35] Davies KP (2009). The role of opiorphins (endogenous neutral endopeptidase inhibitors) in urogenital smooth muscle biology. J. Sex Med..

[36] Kanika ND, Melman A, Davies KP (2010). Experimental priapism is associated with increased oxidative stress and activation of protein degradation pathways in corporal tissue. Int. J. Impot. Res..

[37] Tong Y (2008). The opiorphin gene (ProL1) and its homologues function in erectile physiology. BJU Int..

[38] Al-Saffar MT, Al-Sandook TA, M. Y-Taha, (2013). A possible new concept in the mechanism of action of local anesthesia. Am. J. Med. Biol. Res..

[39] Ruangri S, Jorns TP, Chaiyarit P (2019). Opiorphin level in unstimulated whole saliva of burning mouth syndrome patients. J. Med. Assoc. Thailand.

[40] Page MJ, Bossuyt PM, Boutron I, Hoffmann TC, Mulrow CD (2021). The PRISMA 2020 statement: An updated guideline for reporting systematic reviews. BMJ.

[41] Needleman IG (2002). A guide to systematic reviews. J. Clin. Periodontol..

[42] Tufanaru, C. M. Z., Aromataris, E., Campbell, J., & Hopp, L. Chapter 3: Systematic reviews of effectiveness. In *JBI Manual for Evidence Synthesis* (Aromataris, E., Munn, Z., ed) (JBI, 2020). https://synthesismanual.jbi.global.

[43] Moola, S. M. Z., *et al.* Chapter 7: Systematic reviews of etiology and risk . In *JBI Manual for Evidence Synthesis* (Aromataris, E., & Munn, Z. eds.) (JBI, 2020). https://synthesismanual.jbi.global.

[44] Briggs IJ (2016). JBI Critical Appraisal Checklist for Analytical Prevalence Studies.

[45] Higgins, J., Cochrane Handbook for Systematic Reviews of Interventions Version 5.1.0. http://handbook.cochrane.org/ (The Cochrane Collaboration, 2011).

[46] Parida SK (2018). A study of salivary opiorphin levels using different anesthetic drugs and techniques—a randomized controlled clinical study. J. Stomatol. Oral Maxillofac. Surg..

[47] Boucher Y (2016). Opiorphin levels in fluids of burning mouth syndrome patients: A case–control study. Clin. Oral Invest..

[48] Brkljačić L (2011). Development and validation of a liquid chromatography-tandem mass spectrometry method for the quantification of opiorphin in human saliva. J. Chromatogr. B Analyt. Technol. Biomed. Life Sci..

[49] Accioni F, García-Gómez D, Rubio S (2021). Exploring polar hydrophobicity in organized media for extracting oligopeptides: Application to the extraction of opiorphin in human saliva. J. Chromatogr. A.

[50] Porporatti AL (2023). Is burning mouth syndrome associated with stress? A meta-analysis. J. Oral. Rehabil..

[51] Reaux-Le Goazigo A (2019). Dual enkephalinase inhibitor PL265: A novel topical treatment to alleviate corneal pain and inflammation. Pain.

[52] Mei HR (2023). Efficacy of dual enkephalinase inhibition in a preclinical migraine model is mediated by activation of peripheral delta opioid receptors. Headache.

[53] Dufour E (2013). Opiorphin secretion pattern in healthy volunteers: Gender difference and organ specificity. Biochem. Anal. Biochem..

[54] Asmundson GJ, Katz J (2009). Understanding the co-occurrence of anxiety disorders and chronic pain: State-of-the-art. Depress Anxiety.

[55] Anna K (2021). Salivary biomarkers (opiorphin, cortisol, amylase, and IgA) related to age, sex, and stress perception in a prospective cohort of healthy schoolchildren. Mediat. Inflamm..

[56] Turk DC (2008). Identifying important outcome domains for chronic pain clinical trials: An IMMPACT survey of people with pain. Pain.

