# Supplementary material for: Opiorphin as a biomarker of orofacial conditions: a meta-analysis
Source: Sci Rep. 2023 Sep 19;13:15533. doi: 10.1038/s41598-023-42051-y (PMC10509258; doi:10.1038/s41598-023-42051-y)
Supplement: Supplementary file 1 — Supplementary Information. [file 41598_2023_42051_MOESM1_ESM.docx]

**Appendix 1** - Database search strategy (June 1^st^ 2021 and Updated October 28^th^ 2022)

| **Database** | **Search** |
| --- | --- |
| **PubMed** | opiorphin OR opiorphine OR opiorfhine OR sialorphin OR "SMR pentapeptide" |
| **Scopus** | opiorphin OR opiorphine OR opiorfhine OR sialorphin OR "SMR pentapeptide" |
| **Cochrane** | opiorphin OR opiorphine OR opiorfhine OR sialorphin OR "SMR pentapeptide" |
| **Web of Science** | opiorphin OR opiorphine OR opiorfhine OR sialorphin OR "SMR pentapeptide" |
| **LILACS** | opiorphin OR opiorphine OR opiorfhine OR sialorphin OR "SMR pentapeptide" |
| **EMBASE** | opiorphin OR opiorphine OR opiorfhine OR sialorphin OR "SMR pentapeptide" |
| **LIVIVO** | opiorphin OR opiorphine OR opiorfhine OR sialorphin OR "SMR pentapeptide" |
| **Google Scholar** | (opiorphin OR opiorphine OR opiorfhine) filetype:pdf |
| **OpenGrey** | opiorphin |
| **ProQuest** | opiorphin OR opiorphine OR opiorfhine OR sialorphin OR "SMR pentapeptide" |

**Appendix 2 -** Articles excluded and the reasons for exclusion (n=14).

| **Author, year** | **Reasons for exclusion*** |
| --- | --- |
| 1. Balasubramanian S, 2013 [44] | 2 |
| 2. Brkljacic L, 2011[38] | 2 |
| 3. Dahan A, 2017 [45] | 4 |
| 4. Dufour E, 2013 [40] | 2 |
| 5. Heubach N, 2005 [46] | 4 |
| 6. Ibrahim A, 2018 [47] | 4 |
| 7. Rougeot C, 2007 [48] | 1 |
| 8. Ruangsri S, 2019 [49] | 1 |
| 9. Sabalic M, (No year) [50] | 4 |
| 10. Salaric I, (2021) [51] | 3 |
| 11. Thierauf J, 2016 [52] | 3 |
| 12. Thomsen AB, 2020 [53] | 4 |
| 13. Van Elstraete A, (No year) [54] | 1 |
| 14. Wu Q, 2019 [55] | 2 |

Legend: 1) Studies in animals; 2) Studies where no orofacial condition was evaluated; 3) Studies where opiorphins where not evaluated through saliva, blood, or urine tests; 4) Literature reviews, intervention studies, abstracts, books, letters, case reports (<10 cases) and personal opinions.

**REFERENCES FOR APPENDIX 2**

1. Balasubramanian, S., *Yogic Breathing Changes Salivary Components*. 2013: ClinicalTrials.gov Identifier: NCT02108769.

2. Brkljačić, L., et al., *Development and validation of a liquid chromatography-tandem mass spectrometry method for the quantification of opiorphin in human saliva.* J Chromatogr B Analyt Technol Biomed Life Sci, 2011. **879**(32): p. 3920-6.

3. Dahan, A., *Potent Opioid Analgesia without Respiratory Depression: Could It Be Possible?* Anesthesiology, 2016. **125**(5): p. 841-843.

4. E, D., et al., *Opiorphin Secretion Pattern in Healthy Volunteers: Gender Difference and Organ Specificity*. 2013: Biochem Anal Biochem 2: 136. doi:10.4172/2161-1009.1000136.

5. Heubach, N., C. Wohllebe, and K. Nieber, *Enkephalinase inhibitors - Potent drugs for various indications.* Pharmazeutische Zeitung, 2005. **150**(43): p. 16-21.

6. Ibrahim, A.S., *Serum Opiorphin Level After Intraoperative Intravenous Lidocaine Infusion*. 2018: ClinicalTrials.gov Identifier: NCT03502395.

7. Rougeot, C. and M. Messaoudi, *Identification of human opiorphin, a natural antinociceptive modulator of opioid-dependent pathways.* Medecine/Sciences, 2007. **23**(1): p. 37-39.

8. Ruangsri, S., T.P. Jorns, and P. Chaiyarit, *Opiorphin level in unstimulated whole saliva of burning mouth syndrome patients.* Journal of the Medical Association of Thailand, 2019. **102**(4): p. 63-67.

9. Sabalic, M., et al., *Salivary opiorphin as a potential marker of oral disease.* Oral Diseases. **18**: p. 21-21.

10. Salarić, I., et al., *Salivary melatonin in oral squamous cell carcinoma patients.* Sci Rep, 2021. **11**(1): p. 13201.

11. Thierauf, J., et al., *Expression of Submaxillary Gland Androgen-regulated Protein 3A (SMR3A) in Adenoid Cystic Carcinoma of the Head and Neck.* Anticancer research, 2016. **36**(2): p. 611-615.

12. Thomsen, A.B., et al., *Salivary levels of opiorphin in response to acute short-lasting pain: An experimental study in humans.* European Journal of Neurology, 2020. **27**: p. 1284.

13. Van Elstraete, A., et al., *The Opiorphin Analog STR-324 Decreases Sensory Hypersensitivity in a Rat Model of Neuropathic Pain.* Anesth Analg. **126**(6): p. 2102-2111.

14. Wu, Q., et al., *Screening and identification of biomarkers associated with clinicopathological parameters and prognosis in oral squamous cell carcinoma.* Exp Ther Med, 2019. **18**(5): p. 3579-3587.

**Appendix 3.A –** JBI Critical Appraisal Tools to assessed risk of bias in case-control studies

| **Author, Year** | **Alajbeg I, 2021** | **Boucher Y, 2016** | **Nejad N, 2020** | **Ozdogan S, 2020** | **Saláric I, 2016** |
| --- | --- | --- | --- | --- | --- |
| 1. Were the groups comparable other than the presence of disease in cases or the absence of disease in controls? | Y | Y | Y | Y | Y |
| 1. Were cases and controls matched appropriately? | Y | Y | Y | Y | Y |
| 1. Were the same criteria used for identification of cases and controls? | Y | Y | Y | Y | Y |
| 1. Was exposure measured in a standard, valid and reliable way? | Y | Y | Y | Y | Y |
| 1. Was exposure measured in the same way for cases and controls? | Y | Y | Y | Y | Y |
| 1. Were confounding factors identified? | U | Y | Y | U | Y |
| 1. Were strategies to deal with confounding factors stated? | U | Y | Y | N | Y |
| 1. Were outcomes assessed in a standard, valid and reliable way for cases and controls? | Y | Y | Y | Y | Y |
| 1. Was the exposure period of interest long enough to be meaningful? | Y | Y | U | Y | Y |
| 1. Was appropriate statistical analysis used? | U | Y | Y | Y | Y |
| **Total** | 7Y / 3U | 10Y | 9Y/1U | 8Y / 1N / 1 U | 10Y |

**Appendix 3.B –** JBI Critical Appraisal Tools to assess risk of bias in Critical Appraisal Tool for quasi-experimental studies.

| **Author, Year** | **Al-Saffar M, 2013** | **Ozdogan M, 2019** |
| --- | --- | --- |
| 1. Is it clear in the study what is the ‘cause’ and what is the ‘effect’ (i.e. there is no confusion about which variable comes first)? | Y | Y |
| 1. Were the participants included in any comparisons similar? | Y | Y |
| 1. Were the participants included in any comparisons receiving similar treatment/care, other than the exposure or intervention of interest? | Y | Y |
| 1. Was there a control group? | N | N |
| 1. Were there multiple measurements of the outcome both pre and post the intervention/exposure? | N | Y |
| 1. Was follow up complete and if not, were differences between groups in terms of their follow up adequately described and analyzed? | Y | Y |
| 1. Were the outcomes of participants included in any comparisons measured in the same way? | U | Y |
| 1. Were outcomes measured in a reliable way? | U | Y |
| 1. Was appropriate statistical analysis used? | Y | Y |
| Total | 5Y / 2N / 2U | 8Y / 1N |

**Appendix 3.C –** JBI Critical Appraisal Tools to assess risk of bias in Critical Appraisal Tool for randomized clinical trials.

| **Author, Year** | **Parida S, 2017** |
| --- | --- |
| 1. Was true randomization used for assignment of participants to treatment groups? | U |
| 1. Was allocation to treatment groups concealed? | U |
| 1. Were treatment groups similar at the baseline? | Y |
| 1. Were participants blind to treatment assignment? | U |
| 1. Were those delivering treatment blind to treatment assignment? | U |
| 1. Were outcomes assessors blind to treatment assignment? | U |
| 1. Were treatment groups treated identically other than the intervention of interest? | Y |
| 1. Was follow up complete and if not, were differences between groups in terms of their follow up adequately described and analyzed? | Y |
| 1. Were participants analyzed in the groups to which they were randomized? | Y |
| 1. Were outcomes measured in the same way for treatment groups? | Y |
| 1. Were outcomes measured in a reliable way? | Y |
| 1. Was appropriate statistical analysis used? | Y |
| 1. Was the trial design appropriate, and any deviations from the standard RCT design (individual randomization, parallel groups) accounted for in the conduct and analysis of the trial? | U |
| Total | 7Y / 6U |

**REFERENCES FOR APPENDIX 3**

3.A: Moola S, Munn Z, Tufanaru C, Aromataris E, Sears K, Sfetcu R, Currie M, Qureshi R, Mattis P, Lisy K, Mu P-F. Chapter 7: Systematic reviews of etiology and risk . In: Aromataris E, Munn Z (Editors). JBI Manual for Evidence Synthesis. JBI, 2020. Available from <https://synthesismanual.jbi.global>

3.B: Tufanaru C, Munn Z, Aromataris E, Campbell J, Hopp L. Chapter 3: Systematic reviews of effectiveness. In: Aromataris E, Munn Z (Editors). JBI Manual for Evidence Synthesis. JBI, 2020. Available from <https://synthesismanual.jbi.global>

3.C: Tufanaru C, Munn Z, Aromataris E, Campbell J, Hopp L. Chapter 3: Systematic reviews of effectiveness. In: Aromataris E, Munn Z (Editors). JBI Manual for Evidence Synthesis. JBI, 2020. Available from <https://synthesismanual.jbi.global>

**Appendix 4:** Relative percentages between different groups

| **Group** | **Study** | **Subgroup (Orofacial Condition)** | **Orofacial Condition** | **SD** | **Total** | **Control** | **SD** | **Total** | **Relative Percentage** | **Relative SD** |
| --- | --- | --- | --- | --- | --- | --- | --- | --- | --- | --- |
| **CHRONIC OROFACIAL GROUP (STIMULATED SALIVA)** | Boucher Y, 2016 | BMS (SS) | 28.8 | 25.3 | 21 | 31.1 | 29.1 | 21 | -7.99 | 87.85 |
|  | Saláric I, 2016 | BMS (SS) | 5.819 | 3.594 | 29 | 4.992 | 3.212 | 29 | 14.21 | 61.76 |
|  | Alajbeg I, 2021 | TMD | 1.89 | 1.299 | 11 | 0.646 | 0.467 | 14 | 65.82 | 68.73 |
|  | Nejad N, 2020 | OPMDs (BMS) | 9.409 | 2.369 | 20 | 7.108 | 2.535 | 20 | 24.46 | 25.18 |
|  |  |  |  |  |  |  |  | **Mean Group** | **24.13** | **60.88** |
| **CHRONIC OROFACIAL GROUP (UNSTIMULATED SALIVA)** | Boucher Y, 2016 | BMS (US) | 37.8 | 42.5 | 21 | 67.6 | 188.9 | 21 | -78.84 | 112.43 |
|  | Saláric I, 2016 | BMS (US) | 8.129 | 6.445 | 29 | 5.017 | 2.585 | 29 | 38.28 | 79.28 |
|  | Alajbeg I, 2021 | TMD | 1.89 | 1.299 | 11 | 0.646 | 0.467 | 14 | 65.82 | 68.73 |
|  | Nejad N, 2020 | OPMDs (BMS) | 9.409 | 2.369 | 20 | 7.108 | 2.535 | 20 | 24.46 | 25.18 |
|  |  |  |  |  |  |  |  | **Mean Group** | **12.43** | **71.41** |
|  | **Study** | **SubGroup** | **Before** | **SD** | **Total** | **After** | **SD** | **Total** |  |  |
| **SUSTAINED PAIN GROUP** | Ozdogan M, 2019 | SAP (Before&After) | 28.78 | 5.81 | 24 | 18.58 | 1.85 | 24 | 35.44 | 20.19 |
|  | Ozdogan M, 2019 | SIP (Before&After) | 37.66 | 6.15 | 15 | 18.74 | 1.15 | 15 | 50.24 | 16.33 |
|  | Nejad N, 2020 | Painful Oral Soft-tissue Conditions | 8.268 | 2.414 | 20 | 7.108 | 2.535 | 20 | 14.03 | 29.20 |
|  |  |  |  |  |  |  |  | **Mean Group** | **33.24** | **21.90** |
|  | **Study** | **SubGroup** | **Before** | **SD** | **Total** | **After** | **SD** | **Total** |  |  |
| **ACUTE PAIN AFTER ANESTHESIA GROUP** | Al Saffar, 2013 | Local Anesthesia | 5.96 | 5.38 | 22 | 14.49 | 3.66 | 22 | -143.12 | 90.27 |
|  | Parida S, 2017 | Before&After Anesthesia (10 minutes) | 4.8 |  | 48 | 4.6 |  | 48 | 4.17 |  |
|  |  |  |  |  |  |  |  | **Mean Group** | **-69.48** |  |
|  | **Study** | **SubGroup** | **Before Stimuli** | **SD** | **Total** | **After**  **Stimuli** | **SD** | **Total** |  |  |
| **STIMULATED GROUP** | Ozdogan S, 2020 | Corneal Foreign Body (Tears) | 0.109 | 0.037 | 34 | 0.134 | 0.06 | 32 | -22.94 | 27.61 |
|  | Alajbeg I, 2021 | TMD After Capsaicin (20 minutes) | 1.89 | 1.299 | 11 | 2.277 | 2.052 | 11 | -20.48 | 68.73 |
|  |  |  |  |  |  |  |  | **Mean Group** | **-21.71** | **48.17** |

**Appendix 5:** Summary of the overall strength of evidence using Grading of Recommendations Assessment, Development and Evaluation (GRADE).

| **Certainty assessment** | | | | | | | **№ of patients** | | **Effect** | | **Certainty** | **Importance** |
| --- | --- | --- | --- | --- | --- | --- | --- | --- | --- | --- | --- | --- |
| **№ of studies** | **Study design** | **Risk of bias** | **Inconsistency** | **Indirectness** | **Imprecision** | **Other considerations** | **[intervention]** | **[comparison]** | **Relative (SD)** | **Absolute (95%CI)** |  |  |
| **Chronic Group** | | | | | | | | | | | | |
| 4 | observational studies | serious^a^ | serious^b^ | not serious | serious^c^ | No | 81 cases 84 controls | | 24.13%  (60.88) | 0.62(0.02; 1.22) | VERY LOW^e^ | IMPORTANT |
| **Sustained Group** | | | | | | | | | | | | |
| 3 | observational studies | serious^d^ | serious^b^ | not serious | serious^c^ | No | 59 cases 59 controls | | 32.24% (21.90) | 2.24 (0.34; 4.14) | VERY LOW^e^ | IMPORTANT |
| **Stimulated Group** | | | | | | | | | | | | |
| 2 | observational studies | serious^d^ | not serious | not serious | serious^c^ | NO | 45 cases 43 controls | | 21.71% (48.17) | 0.43 (0.00; 0.85) | VERY LOW^e^ | IMPORTANT |

**SD:** Standard Deviation; **CI:** Confidence Interval

#### Explanations

a. The study of Alajbeg I, 2021 and Nejad N, 2020 presented an unclear risk of bias.

b. I2 presented a value higher than 70%

c. Sample was less than 300 subjects

d. Studies presented an unclear or high risk of bias

e. Any estimate of effect is very uncertain.

**Appendix 6 -** Summary of descriptive characteristics for opiorphin collection.

| **Author**  **Year**  **Country** | **Biological fluids for collection** | **Opiorphin method of collection** | **Information about method of collection** | **Opiorphin method of analysis** |
| --- | --- | --- | --- | --- |
| Alajbeg I, 2021  Croatia.  CLINICAL TRIAL PROTOCOL | Saliva | Opiorphin collection and measurements were performed at three time points, before and twice following the stimulation. Opiorphin levels were quantified by HPLC-MS/MS. | Three saliva samplings - three graded test tubes with trifluoroacetic acid were previously prepared for each participant  The saliva was collected three times: the first time at the beginning of the study, the second time immediately after the end of application of capsaicin-soaked disks (by the end of the 5^th^ minute since the first disk was applied) and the third time 20 minutes after the application was completed (by the end of the 25^th^ minute since the first disk was applied). During the disk set up, participants assessed the pain with the Numerical pain rating scale (NPRS). | HPLC-MS/MS  Electrospray positive ionization-mass spectrometric multiple reaction monitoring (ESI+/MRM) for opiorphin quantification. |
| Al Saffar M, 2013  Iraq | Saliva (Unstimulated) | Rinse with 10 ml of tap water to remove food debris. Then cotton-based techniques, including using a simple cotton dental roll of a specialized device (Salivette), a cotton roll is sucked or chewed in the patient’s mouth for 1 minute, this allows the saliva to be absorbed and collected in the swab in an easy and hygienic fashion, all sample should be collected between 9-11 am and should be clear of blood contamination | Salivette then centrifuged at 3000 rpm for 10 min, and  the clear fluid sample at the bottom of tube is placed in a sterile Eppendorf tube and stored at -20 Ċ to be thawed for analysis. | Measurement of human opiorphin was carried out using an ELISA kit (MyBioSource, USA). The test is a quantitative sandwich enzyme immunoassay technique. Antibody specific for opiorphin has been pre-coated onto a microplate. The test was performed according to the manufacturer’s instructions. |
| Boucher Y, 2016  France | Saliva (Basal and Stimulated) 2 mL,  Blood 30 mL, and  urine 20 mL | ICAReB platform for opiorphin level analyses at the Laboratory of Pharmacology of Pain (Institut Pasteur). | Samples were taken and collected into previously cooled polypropylene tubes (15 or 50 ml, Nunc-VWR, France) containing a mixture of peptidase inhibitors at concentrations to achieve the following final amounts: Aprotinin 1.7 mg/ml or 1000 KIU/ml (Sigma-Aldrich, France), EDTA 1 mM (Fluka), and bestatin 200 μM (Calbiochem). | The competitive-ELISA immunoassay for opiorphin has the following characteristics: IC50 at 30 ± 9 ng/ml and IC85 at 2 ng/ml of opiorphin (mean ± SD) for n = 9 independent inter-day assays, each with 9 points of concentration from 500 to 1.9 ng/ml in duplicate. The immunoassay is specific for opiorphin QRFSR-peptide. The functionally related sialorphin QHNPR-peptide as well as the QRFS, RFSR, and FSR opiorphin-related fragments were not significantly recognized by the antibody (cross-reaction ≤1%). |
| Nejad N, 2020  India | Saliva | Subjects were asked to rinse their mouth with alcohol-free mouthwash, and unstimulated whole saliva (UWS) was collected using a spit method. | The samples were then centrifuged and the supernatant was stored at –80°C till analysis. | Human Opi ELISA kit (Wuhan Fine Biotech Co., Ltd.), a competitive ELISA (enzyme-linked immunosorbent assay) kit, with a detection range of 0.156–10 ng/mL and sensitivity <0.094 ng/mL. |
| Ozdogan M, 2019  Turkey | Saliva | Unstimulated whole saliva was collected from all patients before starting the endodontic treatment, at 7 days after the treatment and at 30 days after the treatment. | A saliva collection kit was devised using dental cotton rolls, 15ml polypropylene centrifuge tubes (Fisher Scientific, MA, USA) and 1ml pipette tips with aerosol barriers (Eppendorf, Hamburg, Germany). The pipette tips were placed inside the centrifuge tubes and the cotton rolls were placed on top of the pipette tips. The kits were autoclaved and kept sterile until they were used. Before specimen collection, the patients were asked to thoroughly wash their mouths with water. Then they were given a cotton roll and asked to slowly chew the roll for 60 s while rotating it inside the whole mouth. Then the cotton roll was placed back into the tube, on the pipette tip. The specimens were stored at −80 °C until the time of analysis.  The frozen specimens were thawed at 4 °C. The saliva was extracted from the cotton rolls by centrifuging at 1000 rcf for 5 min. During centrifugation, the saliva passed from the aerosol barrier of the pipette tip, filtering out any particulates or contaminants, and was collected at the bottom of the tube. Approximately 2 ml of saliva was obtained from each specimen collection. 1 ml of saliva was used for measurements from each specimen and the remaining specimens were stored at −80 °C | A human opiorphin ELISA kit (Hangzhou Eastbiopharm Co., Ltd., Hangzhou, China) was used to measure the opiorphin levels in saliva. The ELISA kit uses the quantitative sandwich enzyme immunoassay technique. The tests were performed following the manufacturer’s instructions using an ELx50 automated microplate strip washer (BioTek Instruments Winooski, VT, USA) and ELx800 microplate reader (BioTek Instruments Winooski, VT, USA). |
| Ozdogan S, 2020  Turkey | Tears | Collected with 50-mL capillary tubes until the tubes were filled. | The tubes were transferred immediately to a -80°C freezer and kept there until analyzed. | A human opiorphin enzyme-linked immunosorbent assay (ELISA) kit (Hangzhou Eastbiopharm Co, Ltd, Hangzhou, China) was used to measure the opiorphin levels in tear. This ELISA kit uses the quantitative sandwich enzyme immunoassay technique. An automated ELISA washer (Awernes Starfax 2600) was used for the ELISA method. Opiorphin levels were measured on an ELISA reader (Awareness Chromate 4300; Awareness Stat Fax 2600, FL). |
| Parida S, 2017  India | Saliva | Salivary samples were collected twice from each patient. The first sample was collected after thorough clinical examination from patients before injecting the local anaesthetic.  The second sample was collected 10 minutes after injecting the local anestehtic from the same patient. Both samples were unstimulated salivary samples, and were collected using a passive drool technique from the floor of the mouth. | The salivary samples were transferred into graduated polypropylene tubes (Tarsons products Pvt Ltd), after being labeled. | Estimation of human opiorphin was done using an ELISA Kit (Sincere Biotech, China). This was an in-vitro quantitative test.  The stored salivary samples were centrifuged at 3000 rpm for 10 minutes. |
| Saláric I, 2016  Croatia | Saliva | Subjects abstained from food and beverages at least for 8 h and did not brush their teeth prior to sample collection. Saliva sampling took place in the morning, between 8 and 10 a.m.  Chromatography-mass spectrometry was utilized for quantification of salivary opiorphin. | Saliva was obtained from the mouth floor with a vacuum saliva collector that delivered it into a pre-weighed graduated tube containing 300 μl of trifluoroacetic acid (TFA) kept on ice. Salivation was induced by 1 % ascorbic acid solution by applying it onto the antero- dorso-lateral surfaces of the tongue and vestibular mucosa using a cotton swab for 60 s, followed by a mouth rinse using the rest of the ascorbic acid solution for 30 s. Afterwards, the samples were vortexed and left on ice for 20 min and afterwards centrifuged (20,000×g, 30 min, at 4 °C). Supernatant (800 μl) was placed into a separate tube and then freeze-dried. The residue was dissolved in 200 μl of 0.1 % formic acid in water, and an aliquot of 30 μl was analyzed by LC-MS/MS (Agilent Technologies 1200 series HPLC system, Agilent Technologies Inc., Palo Alto, CA, USA). | Electrospray positive ionization-mass spectrometric multiple reaction monitoring (ESI+/MRM) experiments were used for quantifying opiorphin. Agilent MassHunter software was used for data processing. Compound-specific MRM was performed with ESI-MS/MS to ensure the quantification accuracy. |

Legend: ELISA: Enzyme Linked ImmunonoSorbent Assay; SWS: Stimulated Whole Saliva; UWS: Unstimulated Whole Saliva.
